# Prognostic Value of a Three-DNA Methylation Biomarker in Patients with Soft Tissue Sarcoma

**DOI:** 10.1155/2020/8106212

**Published:** 2020-05-15

**Authors:** Yuxiao Chen, Rui Zhu, Min Chen, Wenna Guo, Xin Yang, Xin-Jian Xu, Liucun Zhu

**Affiliations:** ^1^Department of Mathematics, Shanghai University, Shanghai 200444, China; ^2^School of Life Sciences, Shanghai University, Shanghai 200444, China; ^3^School of Life Sciences, Zhengzhou University, Zhengzhou, Henan 450001, China

## Abstract

Soft tissue sarcomas (STS) are a highly aggressive and heterogeneous group of malignant mesenchymal tumors. The prognosis of patients with advanced or metastatic STS remains poor, and the main therapy of STS patients combines primary surgery, radiotherapy, and chemotherapy. Aberrant DNA methylation shows close association with the pathogenesis and tumor progression. Therefore, DNA methylation biomarkers might have the potential in accurately predicting the survival of STS patients. In order to identify a prognostic biomarker based on DNA methylation sites, a comprehensive analysis of the DNA methylation profile of STS patients in the Cancer Genome Atlas (TCGA) database was performed. All samples were randomly divided into training and testing datasets. Cox proportional hazards regression analysis was performed to identify a prognostic biomarker that contains three DNA methylation sites. The Kaplan–Meier analysis demonstrated that the 3-DNA methylation biomarker discriminated patients into high-risk and low-risk groups, both in the training and in the testing datasets, and the area under the receiver operating characteristic curve values (AUCs) were 0.844 (*P* < 0.001, 95% CI: 0.740–0.948) and 0.710 (*P* = 0.002, 95% CI: 0.595–0.823), respectively. Besides, this biomarker presented superior prognostic performance in STS patients with different age, sex, tissue of origin, therapy, and histologic subtypes. Compared with other prognostic biomarkers, this biomarker tended to be a more precise prognostic factor in STS patients. Moreover, methylation sites in this biomarker might provide a new way for clinicians to make decisions regarding the intervention and assess the effectiveness of an individual therapeutic strategy.

## 1. Introduction

Soft tissue sarcomas (STS) are a heterogeneous group of malignant tumors that mainly arise from mesenchymal stem cells which can present in various parts of the human body, including the extremities, trunk, and retroperitoneum [1]. More than 50 tumor subtypes that are relevant to STS have been identified [2]. According to the recent statistics, the STS cases might reach an estimated number of 12,750 and 5,270 deaths worldwide in 2019, accounting for 0.7% of all cancer cases and 0.9% of cancer deaths separately [3]. The survival rate and prognosis of patients with STS remain very disappointing. The 5-year survival rate of STS patients is about 50%, showing no improvements over the years [4]. The median survival is only 15 months, especially in patients with lung metastases [5]. For most tumors, risk stratification and targeted therapy can significantly improve the therapeutic effects, and researches on STS show similar results [6]. Surgery is often considered curative for localized STS, radiotherapy can degrade the risk of local recurrence, and chemotherapy is usually reserved for managing metastatic patients [7–9]. These studies have indicated that it is important to stratify the risks of STS patients accurately and select appropriate therapeutic modalities for patient management in the future. However, the current research studies in this field, which are referred to as prognostic researches, are relatively less and limited to focus on a specific STS histologic subtype that is of no help to the risk stratification of most STS patients [10–12].

In recent years, DNA methylation has been widely regarded as a prognostic biomarker with great potential. Aberrant DNA methylation plays a crucial role in cancer pathogenesis and progression [13]. Hypermethylation of the promotor regions inhibits certain tumor suppressor genes, which is considered as a shared trait of many malignant tumors [14]. Genetic and nongenetic factors shape DNA methylation collectively, which can reflect the cumulative exposure of patients towards some risk factors such as transgenerational inheritance, in utero environment, obesity, smoking, age, chronic inflammation, etc. [15]. Therefore, DNA methylation markers might aid in deducing tumor pathogenesis and progression. Furthermore, DNA methylation markers are more stable when compared with RNA- and protein-based markers [16]. Due to these advantages, more and more studies have illustrated the potentiality of DNA methylation as a prognostic marker in cancers. For example, in renal cell carcinoma, *GATA5* hypermethylation is frequently presented and shows a significant association with shortened progression-free survival [17]. Hypomethylation of long interspersed nucleotide element-1 (*LINE-1*) is unfavorable with regard to the prognosis of colorectal cancer in patients [18]. Some scholars have conducted studies on STS as well. Hypermethylation of *RASSF1A* promoter is associated with shorter duration of survival in patients with stage II and III STS [19]. DNA hypermethylation in *p*16^(*INK*4*a*)^ gene promoter can lead to the shorter survival in patients with malignant STS [20]. However, most of the STS methylation analyses involve a small number of samples or merely focus on the methylation of a single specific gene.

The Cancer Genome Atlas (TCGA) is a large-scale and open-access data platform that provides 269 soft tissue sarcoma samples and a corresponding DNA methylation profile. To obtain a more systematic and comprehensive understanding of how DNA methylation takes part in the pathogenesis and development of STS, the genome-wide DNA methylation profile of STS samples in TCGA was analyzed. As a consequence, a 3-DNA methylation prognostic biomarker was identified with multivariate Cox regression analysis. The receiver operating characteristic (ROC) curve and Kaplan–Meier analysis were performed to show the performance of this biomarker in predicting the survival of STS patients. The predictive utility of this biomarker was further validated by regrouping patients with different clinical characteristics. Meanwhile, the prognostic performance of the 3-DNA methylation biomarker with other known molecular biomarkers was compared. Furthermore, we analyzed the biological function and the latent impacts of these methylation sites on the pathogenesis and development of STS, and a few recommendations directed at the therapy of STS patients were proposed.

## 2. Materials and Methods

### 2.1. Patients and DNA Methylation Data from TCGA

**Figure 1:**
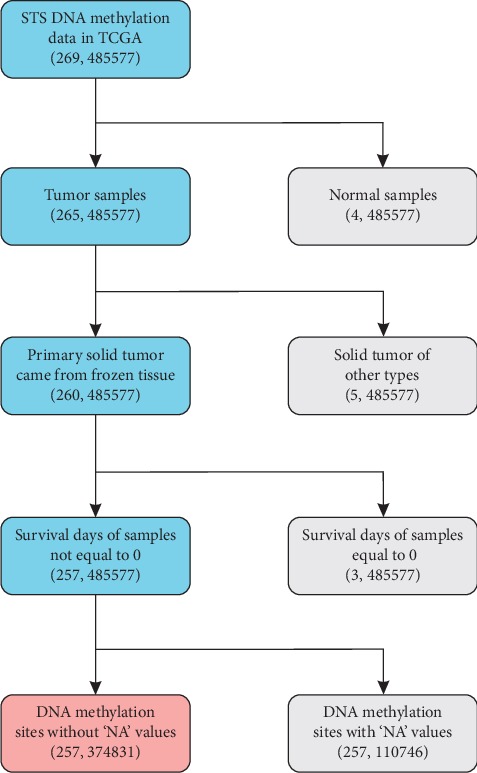
Data cleaning steps for STS DNA methylation data. In the bracket of each box, the number of samples was on the left and the number of methylation sites on the right. The total data of 269 STS samples and 485577 methylation sites were downloaded from the TCGA database. First of all, 4 normal samples were removed, as this study aimed to investigate the prognosis of STS patients. Among the remaining 265 tumor samples, 5 other types of solid tumor samples were dropped, which included 3 recurrent solid tumor samples, 1 metastatic solid tumor sample, and 1 primary solid tumor sample obtained from formalin-fixed paraffin-embedded tissue that was proven to be ineffective for sequencing analysis. As a result, 260 primary solid tumor samples were retained. To avoid possible misunderstandings on the follow-up survival time of STS patients, 3 samples with a survival time of 0 days were excluded. Meanwhile, all the DNA methylation sites containing 'NA' values were removed. Finally, a total of 257 samples with clinical information and 374831 DNA methylation sites were preserved for subsequent analysis.

Clinical information of 269 STS patients and relevant DNA methylation data involved in this study were downloaded from the TCGA database. The DNA methylation profile was measured based on the Infinium HumanMethylation450 BeadChip. The *β*-values of 485577 DNA methylation sites that represent the ratio between the methylated array intensity and the total array intensity falling between 0 (no methylation) and 1 (full methylation) were listed in each sample. During the process of data cleaning, the samples that satisfied the following criteria were removed: (1) diagnosed as nonsarcoma; (2) nonprimary solid tumors; (3) samples were not obtained from the frozen tissues; and (4) the survival time of patients was equal to 0 days. Meanwhile, the methylation sites containing missing values were excluded. Accordingly, 257 samples with 374,831 methylation sites were retained for subsequent analysis. The histological subtypes of these samples belong to STS (Supplementary 1). It is worth noting that patients are censored if they are alive, and whose survival is recorded as the number of days from the start till the end of follow-up. The entire steps for data cleaning are presented in Figure 1. All samples were randomly grouped, and half of which was used as a training dataset (Supplementary 2) for constructing the prognostic model and the remaining half as a testing dataset (Supplementary 3) for verifying the efficacy of the model.

### 2.2. Statistical Analysis

Statistical analysis involved in this study was based on the open-source software R (version 3.4.4) and Perl (version 18). The *survival* package in R was used for Cox proportional hypothesis test and Cox proportional hazards regression analysis. The ROC analysis was performed to demonstrate the accuracy of the biomarker model in predicting the overall survival (OS) of patients, and the AUCs were calculated by Perl. The Kaplan–Meier analysis was used to clarify the relationship between the prognostic model and the OS of the patient. The above analysis involved some statistical hypothesis tests, in which the likelihood ratio test was used to evaluate the significance of one or more independent variables in Cox regression analysis [21]. Whether the AUC was significantly equal to 0.5 was examined by *Z*-test [22]. The Log-rank test and Breslow test were used to assess significant difference in the cumulative survival rate between patients with high and low risks. The former is sensitive to long-term survival, while the latter is more sensitive to short-term survival [23]. The difference in DNA methylation levels between patients with short-term survival (≤3 years) and long-term survival (>3 years) was tested by the Mann–Whitney *U* test.

### 2.3. Establishment of the Prognostic Biomarker Model

First, the Cox proportional hazards hypothesis test (*P* > 0.05) and univariate Cox proportional hazards regression analysis (*P* < 0.01) were used to screen DNA methylation sites that were significantly associated with the OS of STS patients, and sites in the training dataset that satisfied the requirements were used as candidate markers. Then, all the possible combinations of two, three, four, and five candidate markers were constructed, and multivariate Cox regression analysis was used to further screen these combinations that were significantly associated with patient survival. Finally, according to the *P* values of likelihood ratio test, a 3-DNA methylation biomarker was identified, which was the optimal biomarker when compared with other combinations. The risks of STS patients were stratified with the following risk score formula, constructed by the Cox regression coefficients of the model:

(1) $$\begin{array}{c} \text{risk }{\text{score}}_{i}=\;\overset{n}{\underset{j=1}{\sum }}a_{j}\beta_{i,j}, \end{array}$$

where *i* represents the patients and *n* represents the number of DNA methylation sites in the model; *a*_*j*_ is the Cox regression coefficient of site *j*. *β*_*i*,*j*_ is the methylation level of site *j* in patient *i*. According to formula (1), the risk scores of all patients were calculated, and the median risk score was set as a threshold to classify patients into the low-risk group and high-risk group. Next, the Kaplan–Meier curve and Log-rank test and Breslow test were used to assess the differences regarding the cumulative probability of survival between the two groups, both in the training and testing datasets. Furthermore, the whole dataset was regrouped based on different clinical characteristics such as sex, age, tissues of origin, histological subtypes, and adjuvant therapeutic modalities to further validate the prognostic ability of the signature by performing ROC and Kaplan–Meier analyses.

## 3. Results

### 3.1. Clinical Characteristics of STS Patients

The median age of the 257 STS patients included in this study was 61 (ranging from 20 to 89) and the mean survival was 1189 days (ranging within 15–5723 days). In this dataset, the number of male patients differed from the number of female patients to a lesser extent, which was 117 (45.53%) and 140 (54.47%), respectively. Besides, the locations of STS were various. Most of the samples were originated from the connective soft tissues in head and neck, chest, pelvis, limbs, and trunk (*N* = 114, 44.36%). This category was briefly abbreviated as connective soft tissue. The rest of the samples were originated from the retroperitoneum (*N* = 99, 38.52%), uterus (*N* = 31, 12.06%), and other tissues (*N* = 13, 5.06%) including kidney, stomach, and ovary. According to the histological subtypes, all samples were classified into 39 (15.17%) cases with fibromyxosarcoma, 59 (22.96%) cases with liposarcoma, 104 (40.47%) cases with leiomyosarcoma, 10 (3.89%) cases with synovial sarcoma, 3 (1.17%) cases with giant cell sarcoma, 9 (3.5%) cases with malignant peripheral nerve sheath tumor, and 33 (12.84%) cases with undifferentiated sarcoma. All these 257 patients underwent surgery; among them, 137 (53.31%) cases received adjuvant pharmaceutical therapy and the remaining 120 (46.69%) received adjuvant radiation therapy. The detailed information against clinical characteristics is summarized in Table 1.

### 3.2. Establishment of the 3-DNA Methylation Prognostic Biomarker Model

**Figure 2:**
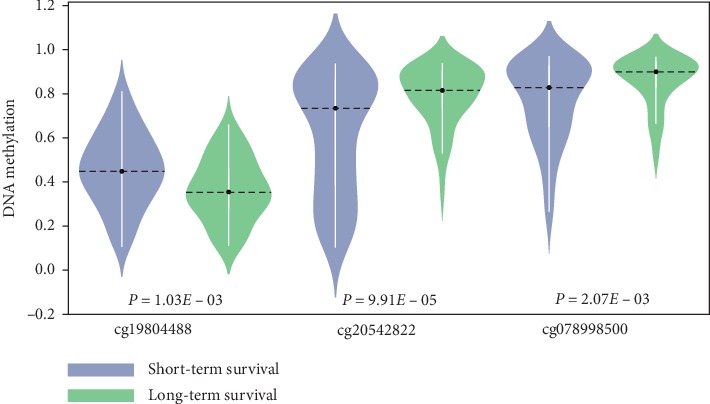
Violin plots of methylation level at a single CpG site for STS patients with short- and long-term survival in the training dataset. Here, short-term survival refers to a survival time that was less than or equal to 3 years, and long-term survival refers to a survival time for more than 3 years. Blue violin plots are for STS patients with short-term survival, and green violin plots are for patients with long-term survival. The dotted line through the black dot represents the median, the range of the white box is from the lower quartile to the upper quartile, the thin white line represents the 95% confidence interval, and the outer shape is the density. The two on the left are violin plots of methylation distribution of cg19804488 for patients with short-term survival and long-term survival. Similarly, the two in the middle correspond to cg20542822, and the two on the right correspond to cg07898500. The Mann–Whitney *U* test showed that there was a significant difference in long-term and short-term patients with respect to the methylation levels of the three methylation sites.

The univariate Cox proportional hazards regression analysis and Cox proportional hazards hypothesis test were used to identify the DNA methylation sites that were significantly associated with the OS of patients with STS in the training dataset. The screen rules were followed by setting the thresholds of the *P* value of the likelihood ratio test (*P* < 0.01) and Cox proportional hazards hypothesis test (*P* > 0.05) in Cox proportional hazards regression analysis. As a result, 15551 sites were retained as candidate markers. Multivariate Cox regression analysis was then performed on candidate markers. Finally, a biomarker model containing three CpG sites (cg19804488, cg20542822, and cg07898500) was selected to analyze the prognosis of patients. According to the Cox regression coefficient of each site in the model, the risk score formula was presented as follows:

(2) $$\begin{array}{c} \text{risk score}=7.537\times \beta_{\text{cg}19804488}-3.301\times \beta_{\text{cg}20542822}-3.825\times \beta_{\text{cg}07898500}, \end{array}$$

where *β*_cg19804488_represents the *β* value of cg19804488, and the same represents the other two sites cg20542822 and cg07898500. Obviously, high DNA methylation level of cg19804488 can result in the increasing risk of STS patients, while high methylation levels of cg20542822 and cg07898500 reduce the risk. The genes corresponding to the three CpG sites are *GALK1* (galactokinase 1), *COL6A5* (collagen type VI alpha 5 chain), and *VCX3B* (variable charge X-linked 3B). Relevant Cox regression coefficients, *P* values, gene symbols, and chromosomal positions are shown in Table 2. Furthermore, DNA methylation levels of the three sites in patients with short- and long-term survival were examined.  Figure 2 explicates that the methylation level of cg19804488 in the group of patients with short-term survival was significantly higher than that in the group of patients with long-term survival (*P* = 1.03*E* − 03). In contrast, both cg20542822 and cg07898500 had a higher methylation level in patients with long-term survival compared with patients with short-term survival; the *P* values were 9.91*E*−05 and 2.07*E*−03 separately. The result was consistent with the Cox regression analysis above.

### 3.3. Prognostic Performance of the 3-DNA Methylation Biomarker in the Training and Testing Datasets

**Figure 3:**
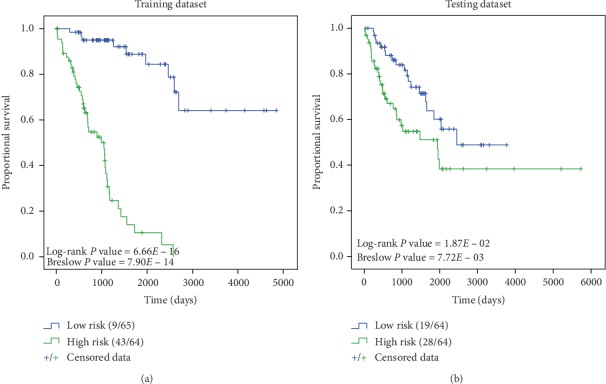
Kaplan–Meier curves showing the ability of the 3-DNA methylation biomarker in distinguishing high and low risk of STS patients. Blue line represents the survival curve of low-risk patients, and green line represents the survival curve of high-risk patients. “+” represents the censored samples. The data on the left side of the brackets represents the number of death samples of the current group, and the right side represents the total number of samples of the current group. (a) Kaplan–Meier curves for high- and low-risk patients in the training dataset (*N*=129). The low risk score in patients was significantly correlated with the better prognosis (Log-rank *P* value = 6.66*E*−16, Breslow *P* value = 7.90*E*−14). (b) Kaplan–Meier curves for high- and low-risk patients in the testing dataset (*N* = 128). A significant difference was shown in the cumulative survival between patients with high and low risks (Log-rank *P* value = 1.87*E*−02, Breslow *P* value = 7.72*E*−03).

A useful prognostic biomarker should be closely related to the OS of patients. The Kaplan–Meier survival analysis was performed to identify the relationship between the 3-DNA methylation biomarker and patient survival in both the training and testing datasets. Based on the above risk score formula (1), the risk score of each patient was calculated. According to the median risk score, patients were divided into the low-risk group and high-risk group, and the Kaplan–Meier curve was drawn. The risk score distribution and median of the two datasets are presented in Supplementary Figure 1. The Log-rank test and Breslow test revealed that the cumulative survival rates of the low-risk group in the training and testing datasets were much higher than those of the high-risk group (Figure 3). At the same time, Kaplan–Meier survival analysis was performed for individual DNA methylation sites on the testing dataset, and neither of which can distinguish high- and low-risk patients (Supplementary Figure 2). This implied that the 3-DNA methylation biomarker exhibited better performance in distinguishing the risks of STS patients than these sites alone as signatures.

**Figure 4:**
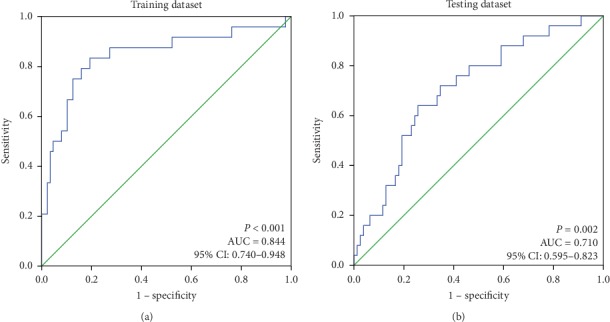
ROC curves showing the prognostic utility of the 3-DNA methylation biomarker. (a) The ROC curve of the 3-DNA methylation biomarker in predicting the OS of STS patients in the training dataset. The AUC is 0.844 (*P* < 0.001, 95% CI: 0.740–0.948). (b) The ROC curve of the 3-DNA methylation biomarker prognostic predictor in the testing dataset. The AUC is 0.710 (*P*=0.002, 95% CI: 0.595–0.823).

To verify the prognostic performance of the 3-DNA methylation biomarker, ROC curve analysis was performed in the training and testing datasets. The AUCs were 0.844 (*P* < 0.001, 95% CI: 0.740–0.948) and 0.710 (*P*=0.002, 95% CI: 0.595–0.823) (Figure 4), respectively. It indicated that the 3-DNA methylation biomarker performed well in predicting the OS of STS patient. Therefore, the 3-DNA methylation biomarker can be used as a prognostic factor for STS patients.

### 3.4. Identifying the Prognostic Performance of the Biomarker by Regrouping the Dataset with Different Clinical Characteristics

**Figure 5:**
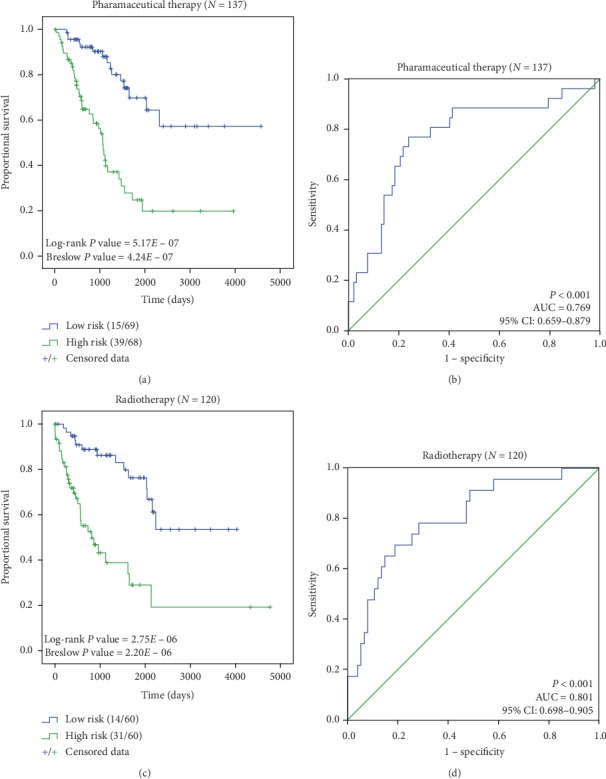
ROC and Kaplan–Meier analyses in groups of patients receiving different adjuvant therapies. (a) and (b) Kaplan–Meier and ROC curves in the group of patients receiving adjuvant pharmaceutical therapy. (c) and (d) Kaplan–Meier and ROC curves in the group of patients receiving adjuvant radiotherapy. It indicates that regardless of the adjuvant treatment therapies the patient received, our biomarker distinguished the high- and low-risk patients significantly.

Clinical characteristics are important indicators for predicting the survival rate in patients with STS [24], which include age, histological subtypes, and tumor locations. Therefore, samples were regrouped based on different clinical characteristics for further validation of the prognostic robustness and independence of the 3-DNA methylation biomarker. The dataset was classified into a group of patients who received adjuvant pharmaceutical therapy and a group of patients who received adjuvant radiation therapy. In the two groups, the median risk scores were −2.174 and −2.233, respectively. Kaplan–Meier analysis showed that the 3-DNA methylation biomarker significantly distinguished the high- and low-risk patients, with the *P* values of Log-rank and Breslow test being less than 0.001 (Figure 5). Moreover, the AUCs were 0.769 (*P* < 0.001, 95% CI: 0.659–0.879) and 0.801 (*P* < 0.001, 95% CI: 0.698–0.905). It suggested that different adjuvant therapies did not impede the prognostic effectiveness of our biomarker. Also, the patients were categorized into two groups by the median age of 61 years as the cut-off point (Supplementary Figure 3). The group of patients under 61 years (*n* = 128, 49.8%) of age and the group of patients older than 61 (*n* = 129, 50.2%) showed a significant difference in the survival rate between high-risk and low-risk patients (*P* < 0.001). The AUCs were 0.734 (*P*=0.002, 95% CI: 0.599–0.870) and 0.815 (*P* < 0.001, 95% CI: 0.721–0.908). In the male and female patient groups, the survival time of the high-risk patient group was also significantly lower than that of the low-risk patient group (Supplementary Figure 4). The AUCs were 0.785 (*P* < 0.001, 95% CI: 0.665–0.915) and 0.773 (*P* < 0.001, 95% CI: 0.676–0.870). For different histological subtypes, taking the limited sample sizes into account, the samples were reallocated into three groups: leiomyosarcoma (*N* = 104, 40.47%), liposarcoma (*N* = 59, 22.96%), and other subtypes of sarcoma (*N* = 94, 36.57%). In the three groups, the AUCs were 0.792 (*P* < 0.001, 95% CI: 0.665–0.920), 0.790 (*P*=0.002, 95% CI: 0.626–0.953), and 0.806 (*P* < 0.001, 95% CI: 0.692–0.919), respectively (Supplementary Figure 5). Furthermore, no matter where the tumor located, including retroperitoneal, connective soft tissue, and other tissues, the Kaplan–Meier and ROC analyses showed that in each group, the prognosis of low-risk patients was better than that of high-risk patients (Supplementary Figure 6). All the above results are summarized in Table 3.

### 3.5. Prognostic Superiority of the 3-DNA Methylation Biomarker as Compared with Other Biomarkers

**Figure 6:**
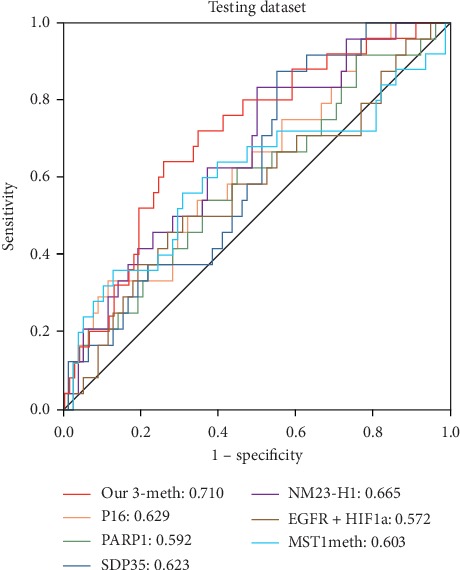
ROC curves of the 3-DNA methylation biomarker compared with other biomarkers that were previously reported. The red bold curve is the ROC curve of the signature established in this research and its AUC is significantly higher than other prognostic markers.

Previous studies have reported several prognostic biomarkers that are correlated with soft tissue sarcomas. For example, Jia et al. explained that high expression of genes *p16* and *NM23-H1* in STS was closely associated with favorable prognosis of patients [25]. *PARP1* expression was considered as a prognostic factor in patients with STS [26]. Low expression of genes *EGFR* and *HIF-1* in STS patients usually contributes to a poor prognosis [27]. The results of Pollino et al. illustrated that increased gene expression of *SDP35* promoted the progression of STS metastasis and could be used as an independent marker of poor prognosis in patients [28]. Hypermethylation of *MST1* showed significant correlation with favorable prognosis in patients with STS [29]. For prognostic biomarkers of STS that were established in the above studies, the prognostic efficiency was assessed and compared with the biomarker identified in this study. The AUCs of these biomarkers are shown in Figure 6 and more information is presented in Supplementary Table 1. It revealed that the prognostic performance of the 3-DNA methylation biomarker was much better than other biomarkers. Notably, the performance of the MST1 methylation biomarker in distinguishing the high- and low-risk STS patients was also assessed. The results are summarized in Table 3. Kaplan–Meier and ROC curves are shown in Supplementary Figures 7–11; they showed that the MST1 methylation biomarker represented poor performance in the group of patients aged over 61 years, the group of patients receiving radiotherapy, and the group of patients with primary lesions of connective soft tissues in the head, neck, chest, etc. Moreover, in all groups, the AUC values of the MST1 methylation biomarker were significantly lower than the 3-DNA methylation biomarker. Therefore, in terms of methylation biomarkers, our 3-DNA methylation biomarker has more advantages.

## 4. Discussion

This study is the first to systematically analyze the genome-wide DNA methylation profile of the STS patients in the TCGA database. The high rate of metastasis and mortality remains a hang-up in most of the STS patients [30]. Therefore, it is of great significance to find prognostic markers for risk stratification and guiding clinicians to scheme therapeutic regimens in STS patients. As a result, a 3-DNA methylation biomarker containing 3 CpG sites (cg19804488, cg20542822, and cg07898500) was identified through univariate and multivariate Cox regression screening. Aberrant methylation of the 3 sites was closely bound up with the survival of STS patients. Kaplan–Meier and ROC analyses showed that this biomarker displayed superior performance in predicting the survival of STS patients. To further confirm the practicability, the dataset was regrouped according to different clinical characteristics such as age, sex, the tissue of origin, etc. In particular, no matter which adjuvant therapy was used, the Kaplan–Meier analysis showed that the 3-DNA methylation biomarker discriminated the survival difference of patients with high and low risks. Besides, the AUCs in all these groups exceeded 0.7. At the same time, compared with other prognostic biomarkers, the AUC of our biomarker was much higher. All the above results indicate that the biomarker is a useful prognostic factor independent of clinical characteristics in STS patients.

More samples and cross-platform datasets could broadly confirm the results of this research which require continuous attention. It is expected that there will be more datasets that can be used to strengthen our conclusions in the future. At the same time, more investigations are required on how the 3-DNA methylation biomarker is involved in the pathogenesis and development of STS.

As mentioned above, in terms of STS, there are few studies on DNA methylation prognostic biomarkers and none of them has been applied in clinical practice so far. However, with the continuous development of DNA methylation detection technology, our research may provide a new direction for clinicians to make decisions regarding interventions and assess the effectiveness of individual therapeutic strategies. Galactose kinase (*GALK1*) is the main enzyme for galactose metabolism. Tang et al. have reported that *GALK1* might be a new target for treating hepatocellular carcinoma (HCC) and also revealed that galactose metabolic pathway exhibited a new posttranscriptional regulation effect on the protein expression of PI3K/AKT signaling pathway in HCC [31]. However, the PI3K/AKT signaling pathway has been proved to be a potential therapeutic target in STS [32, 33]. Therefore, whether *GALK1* could regulate the PI3K signaling pathway through the galactose pathway in patients with STS, thereby promoting the pathogenesis and progression of tumors, should be further studied. Type 6 collagen 5 chain (*COL6A5*) is also known as *COL29A1*, and its variation is closely associated with specific dermatitis [34]. Besides, *COL6A5* acts as a functional ligand of LAIR-1, which can directly inhibit the activation of immune cells. These cells will in turn lose the antitumor response and promote tumor progression. [35]. Immunotherapy is one of the major breakthroughs in oncology, but there is still much controversy going on in STS. Some immune checkpoints including PD-1/PD-L1, CTLA-4, and other blocking therapies only have subtle effects in STS [36]. Perhaps distinct immune targets should be investigated. The possible regulating mechanism of *COL6A5* on LAIR-1 may provide a new strategy. The function of *VCX3B*, a member of the VCX/Y family, has not been clearly described yet. It can be deduced that this gene might increase the stability of some mRNAs, since one of the abilities of *VCX-A*, which has a high sequence similarity with *VCX3B*, is to inhibit mRNA decapping. More gene functions and their association with STS should be evaluated by conducting further experiments.

We are also concerned about the phenomenon that patients with visceral sarcomas and tumors that originate from the viscera often have similar symptoms. For example, some gastrointestinal symptoms such as abdominal pain, weight loss, melasma, or anemia are usually shared between patients with gastrointestinal stromal tumors and common adenocarcinomas. Patients with uterine leiomyosarcoma and common uterine malignancies are both accompanied by painless vaginal bleeding [37]. Therefore, some visceral sarcomas are often managed as primary organ tumors rather than STS [38]. It is excusable if this phenomenon does not interfere with the therapeutic effects of STS patients. However, our research revealed that the therapeutic efficiency of STS in different organs can be predicted by using the same prognostic biomarker. This suggested that visceral sarcomas have something in common that distinguishes them from their tumor counterparts in the primary organ. Accordingly, treating visceral sarcomas differently and carrying out targeted therapy might be an effective new idea.

## 5. Conclusion

Based on the analysis of the genome-wide DNA methylation profile of 257 STS patients in TCGA, a 3-DNA methylation biomarker that was significantly associated with the survival of patients with STS was identified, which likewise embodied the prognostic practicality for STS patients with different age, sex, histologic subtypes, primary organs, etc. Moreover, the 3-DNA methylation biomarker exhibited more advantages in predicting the survival of STS patients when compared with other prognostic markers that were reported previously. Therefore, the biomarker identified in this study can be used as a superior biomarker for the prognosis of patients with STS.

## Figures and Tables

**Table 1:** Clinical characteristics of soft tissue sarcoma patients in the TCGA database.

Characteristics	Groups	Patients					
		Total		Training cohort		Testing cohort	
		(*N* = 257)		(*N* = 129)		(*N* = 128)	
		No.	%	No.	%	No.	%
Sex	Male	117	45.53	59	45.74	58	45.31
	Female	140	54.47	70	54.26	70	54.69

Age at diagnosis	Median	61		60		62	
	Range	20–89		20–89		24–87	
	<61	128	49.80	66	51.16	62	48.44
	≥61	129	50.20	63	48.84	66	51.56

Vital status	Alive	158	61.48	77	59.69	81	63.28
	Dead	99	38.52	52	40.31	47	36.72

Tissue of origin	Uterus	31	12.06	16	12.40	15	11.72
	Connective soft tissue	114	44.36	51	39.54	63	49.22
	Retroperitoneum	99	38.52	54	41.86	45	35.16
	Other	13	5.06	8	6.20	5	3.90

Histological type	FMS	39	15.17	13	10.08	26	20.31
	LPS	59	22.96	30	23.26	29	22.65
	LMS	104	40.47	56	43.41	48	37.50
	SS	10	3.89	6	4.65	4	3.13
	GCS	3	1.17	2	1.55	1	0.78
	MPNST	9	3.50	5	3.87	4	3.13
	US	33	12.84	17	13.18	16	12.50

Adjuvant treatment type	Pharmaceutical	137	53.31	70	54.26	67	52.34
	Radiation	120	46.69	59	45.74	61	47.66

FMS: fibromyxosarcoma; LPS: liposarcoma; LMS: leiomyosarcoma; SS: synovial sarcoma; GCS: giant cell sarcoma; MPNST: malignant peripheral nerve sheath tumor; and US: undifferentiated sarcoma.

**Table 2:** Information about the 3 DNA methylation sites.

Probe ID	Coefficient	*P* value	Gene symbol	Chromosome location
cg19804488	7.537	8.20*E*−07	GALK1	Chr17: 75764282–75764283
cg20542822	−3.301	1.89*E*−08	COL6A5	Chr3: 130379491–130379492
cg07898500	−3.825	2.59*E*−04	VCX3B	ChrX: 8464947–8464948

**Table 3:** Results of Kaplan–Meier and ROC analysis in different groups based on different clinical characteristics.

Regrouping factors	Group	Sample size	Kaplan–Meier Log rank *P* value		Kaplan–Meier Breslow *P* value		AUC		95% CI	
			3-meth	MST1-meth	3-meth	MST1-meth	3-meth	MST1-meth	3-meth	MST1-meth
Sex	Female	140	4.02*E*−07	1.70*E*−02	2.45*E*−07	1.03*E*−02	0.773^*∗*^	0.657	0.676–0.870	0.535–0.779
	Male	117	1.69*E*−05	7.63*E*−03	1.22*E*−05	6.34*E*−03	0.785^*∗*^	0.636	0.665–0.915	0.490–0.782

Age at diagnosis	<61	128	1.10*E*−04	1.87*E*−04	5.26*E*−05	3.13*E*−04	0.734^*∗*^	0.714	0.599–0.870	0.572–0.856
	≥61	129	2.11*E*−06	1.07*E*−01	2.54*E*−07	7.01*E*−02	0.815^*∗*^	0.599	0.721–0.908	0.478–0.720

Tissue of origin	Connective soft tissue	114	2.19*E*−03	3.21*E*−01	8.44*E*−04	2.94*E*−01	0.800^*∗*^	0.483	0.677–0.923	0.299–0.666
	Retroperitoneum	99	9.24*E*−05	1.69*E*−04	6.75*E*−05	3.10*E*−04	0.787^*∗*^	0.701	0.661–0.914	0.574–0.827
	Other tissues	44	8.68*E*−04	1.44*E*−01	6.19*E*−04	1.32*E*−01	0.798^*∗*^	0.692	0.655–0.941	0.504–0.881

Histological type	Leiomyosarcoma	104	3.66*E*−05	1.15*E*−02	3.49*E*−05	1.17*E*−02	0.792^*∗*^	0.657	0.665–0.920	0.502–0.812
	Liposarcoma	59	2.42*E*−04	1.45*E*−04	2.48*E*−04	1.92*E*−04	0.790^*∗*^	0.753	0.626–0.953	0.616–0.890
	Other subtypes	94	7.57*E*−05	4.36*E*−02	3.19*E*−05	3.73*E*−02	0.806^*∗*^	0.579	0.692–0.919	0.402–0.756

Treatment type	Pharmaceutical	137	5.17*E*−07	2.75*E*−04	4.24*E*−07	4.51*E*−04	0.769^*∗*^	0.673	0.659–0.879	0.545–0.800
	Radiation	120	2.75*E*−06	8.08*E*−02	2.20*E*−06	7.25*E*−02	0.801^*∗*^	0.6100	0.698–0.905	0.471–0.750

To be mentioned, 3-meth is the biomarker identified in this paper. The *Z*-test was used to assess whether the AUC of the 3-DNA methylation biomarker is higher than the AUC of the MST1 methylation biomarker. “^*∗*^” represents the *P* value of *Z*-test being less than 0.001, which illustrated that the AUC of our 3-DNA methylation biomarker was significantly higher than the MST1 methylation biomarker.

## Data Availability

This study used public data accessible in The Cancer Genome Atlas (TCGA) database.

## Supplementary Materials

Supplementary Materials Supplementary 1: the 257 sarcoma samples used in this study; their corresponding histological subtypes all belong to soft tissue sarcoma. Supplementary 2: the 129 samples in the training dataset. Supplementary 3: the 128 samples in the testing dataset. Supplementary Table 1: the OS and DNA methylation level or gene expression of the 3-DNA methylation biomarker and other known biomarkers listed above in the testing dataset. Supplementary Figure 1: distribution histograms of the risk score based on the 3-DNA methylation biomarker both in the training and in the testing datasets. Supplementary Figure 2: Kaplan–Meier and ROC analyses of the 3 sites in testing dataset. Supplementary Figure 3: Kaplan–Meier and ROC curves in two groups; grouping is based on their median age 61 at initial diagnosis. Supplementary Figure 4: Kaplan–Meier and ROC curves for STS patients in different sex groups. Supplementary Figure 5: Kaplan–Meier and ROC analyses for STS patients whose tumors belong to different histologic subtypes. Supplementary Figure 6: Kaplan–Meier and ROC analyses for STS patients with tumor originating from different tissues. Supplementary Figures 7–11: Kaplan–Meier and ROC analyses of the MST1 methylation biomarker for STS patients with different clinical characteristics.
