# Supplementary material for: Prognostic Value of a Three-DNA Methylation Biomarker in Patients with Soft Tissue Sarcoma
Source: J Oncol. 2020 May 15;2020:8106212. doi: 10.1155/2020/8106212 (PMC7245661; doi:10.1155/2020/8106212)
Supplement: Supplementary Materials — Supplementary 1: the 257 sarcoma samples used in this study; their corresponding histological subtypes all belong to soft tissue sarcoma. Supplementary 2: the 129 samples in the training dataset. Supplementary 3: the 128 samples in the testing dataset. Supplementary Table 1: the OS and DNA methylation level or gene expression of the 3-DNA methylation biomarker and other known biomarkers listed above in the testing dataset. Supplementary Figure 1: distribution histograms of the risk score based on the 3-DNA methylation biomarker both in the training and in the testing datasets. Supplementary Figure 2: Kaplan–Meier and ROC analyses of the 3 sites in testing dataset. Supplementary Figure 3: Kaplan–Meier and ROC curves in two groups; grouping is based on their median age 61 at initial diagnosis. Supplementary Figure 4: Kaplan–Meier and ROC curves for STS patients in different sex groups. Supplementary Figure 5: Kaplan–Meier and ROC analyses for STS patients whose tumors belong to different histologic subtypes. Supplementary Figure 6: Kaplan–Meier and ROC analyses for STS patients with tumor originating from different tissues. Supplementary Figures 7–11: Kaplan–Meier and ROC analyses of the MST1 methylation biomarker for STS patients with different clinical characteristics. [file 8106212.f1.zip › 8106212.f1/mat.8106212.v2.pdf]

Prognostic value of a three-DNA methylation biomarker in patients with soft tissue sarcoma

Yuxiao Chen, [1] Rui Zhu, [1] Min Chen, [2] Wenna Guo,[3] Xin Yang,[2] Xin-Jian Xu,[1] and Liucun Zhu[2]

[1]Department of Mathematics, Shanghai University, Shanghai, 200444, China
[2]School of Life Sciences, Shanghai University, Shanghai, 200444, China
[3]School of Life Sciences, Zhengzhou University, Zhengzhou, Henan, 450001, China

Correspondence should be addressed to Liucun Zhu; zhuliucun@shu.edu.cn and Xin-Jian Xu; xinjxu@shu.edu.cn

Supplementary Table 1: The AUCs of the 3-DNA methylation biomarker and other known biomarkers in the testing dataset

Signature	AUC	95%CI of AUC	P value	Type
3-DNA methylation	0.710	0.595-0.823	0.002	methylation
p16	0.629	0.504-0.755	0.056	protein coding
PARP1	0.592	0.460-0.724	0.175	protein coding
NM23-H1	0.665	0.546-0.784	0.015	protein coding
SDP35	0.623	0.506-0.741	0.068	protein coding
EGFR+HIF1a	0.572	0.435-0.709	0.287	protein coding
MST1 meth	0.603	0.461-0.745	0.122	methylation

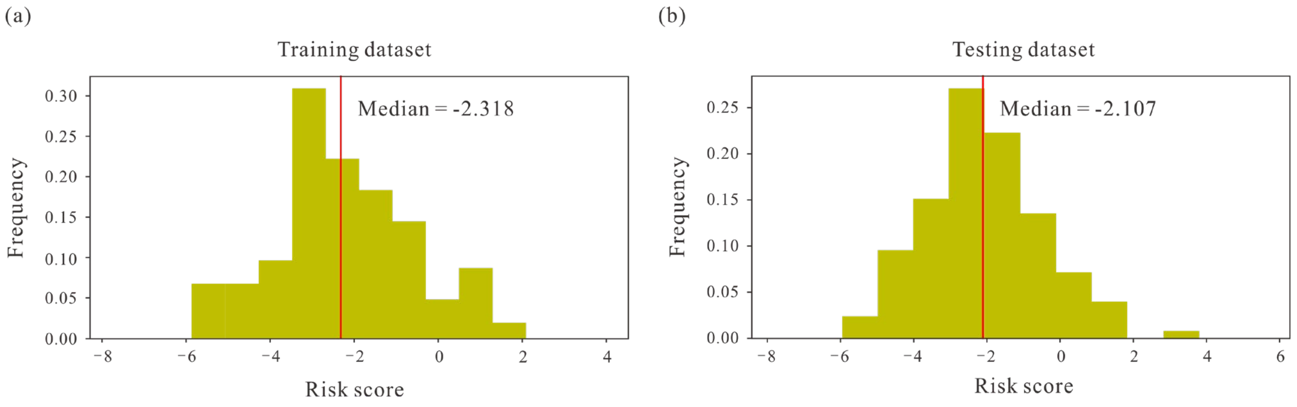

Supplementary Figure 1: Distribution histograms of the risk score based on the 3-DNA methylation biomarker. (a) The distribution histogram which shows the distribution of the risk score in the training dataset, and the red line represents the median score. (b) The distribution histogram which shows the distribution of the risk score in the testing dataset.

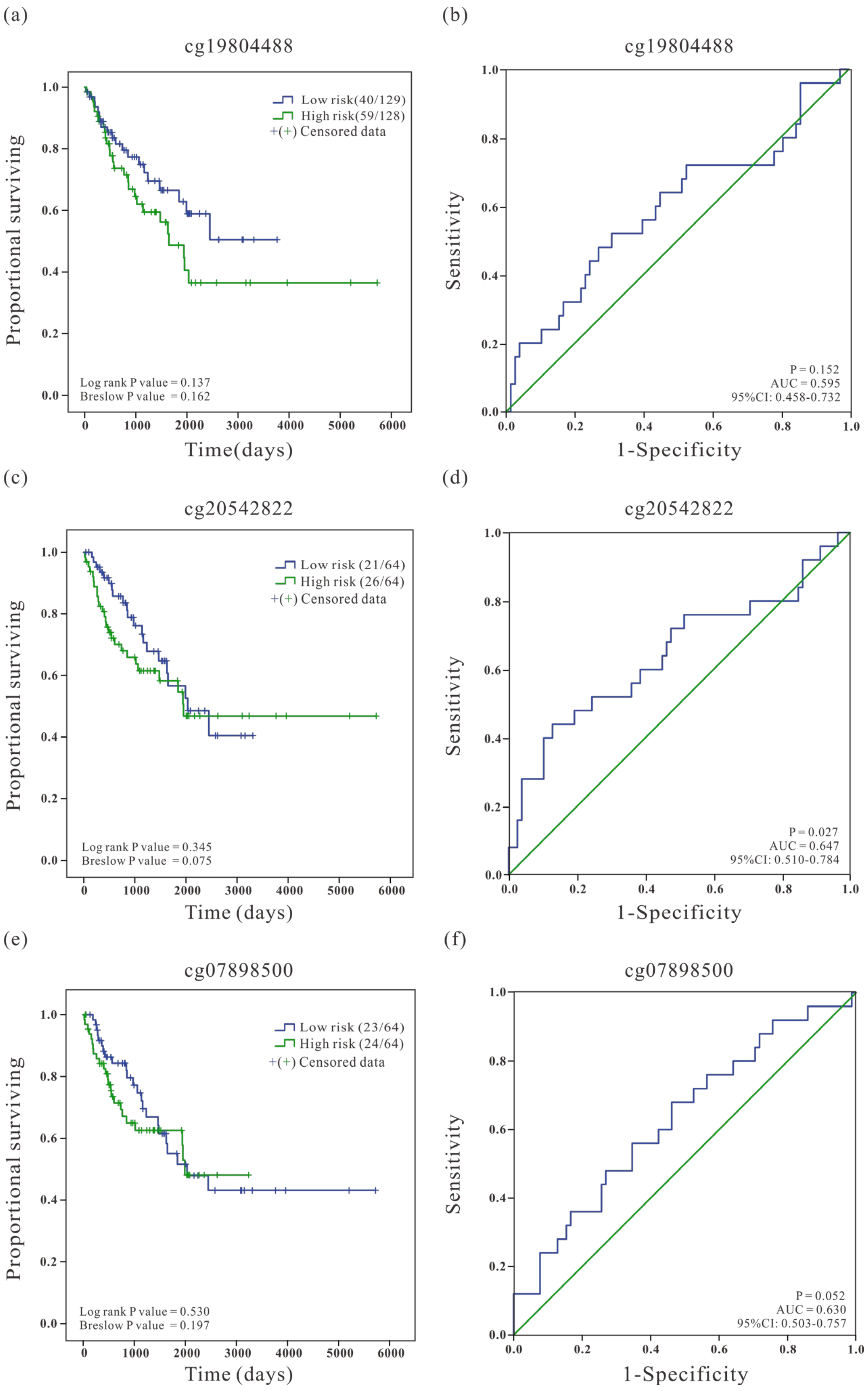

Supplementary Figure 2: Kaplan-Meier and ROC analysis of the 3 sites in testing dataset. (a) The Kaplan-Meier curve of cg19804488 for distinguishing the high- and low-risk STS patients in the testing set. (b) The ROC curve of cg19804488 for exhibiting the predictive sensitivity ang specificity of the survival of STS patients in the testing set. In the same way, (c) and (d) correspond to the Kaplan-Meier and ROC analysis of cg20542822, (e) and (f) correspond to the Kaplan-Meier and ROC

analysis of cg07898500. It showed that all the 3 sites could not distinguish high- and low-risk patients.

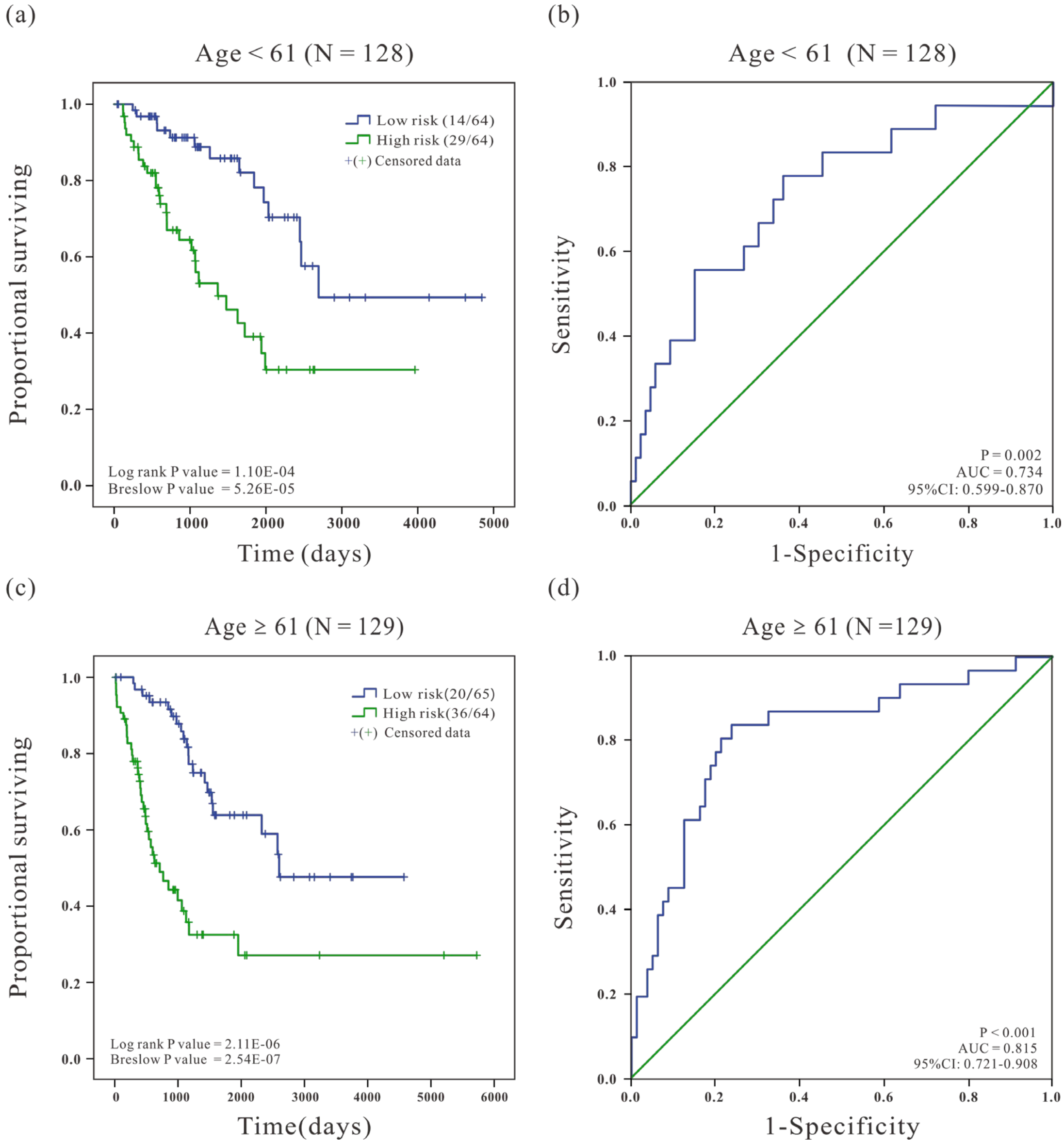

Supplementary Figure 3: Kaplan-Meier and ROC curves in different age groups. (a) Kaplan-Meier curve of the 3-DNA methylation biomarker in the group of patients aged less than or equal to 61, which estimates the survival difference between the low-risk group and the high-risk group. (b) ROC curve of the 3-DNA methylation biomarker exhibits the prognostic performance in the group of patients aged less than or equal to 61. (c) and (d) are the Kaplan-Meier and ROC curves for patients group aged more than 61.

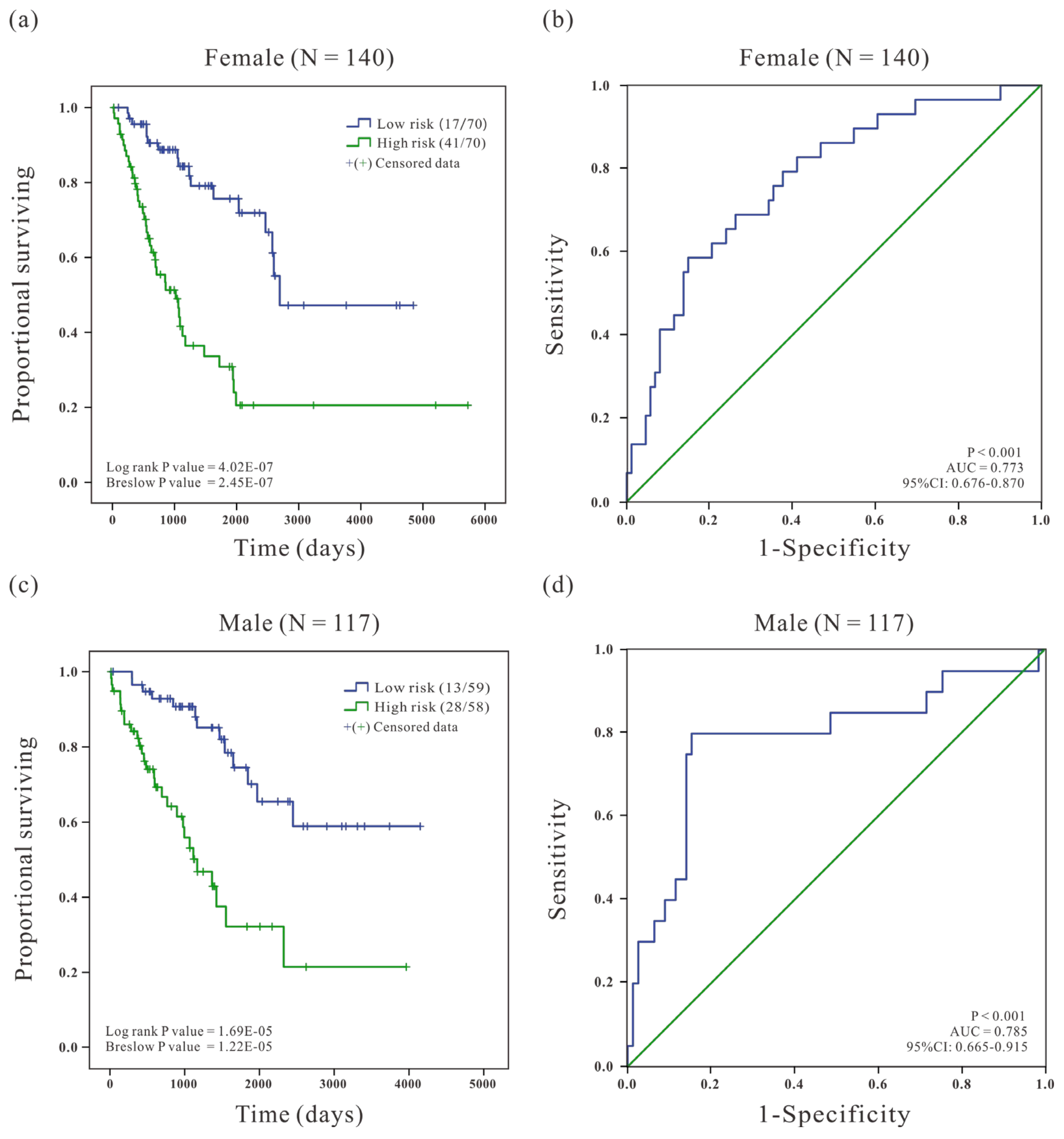

Supplementary Figure 4: Kaplan-Meier and ROC curves for STS patients in different sex groups. (a) The Kaplan-Meier curve estimates the cumulative survival time of STS patients in low- and high-risk group. (b) The ROC curve exhibits the prognostic performance of the 3-DNA methylation biomarker. (c) and (d) Kaplan-Meier and ROC analysis of the biomarker in predicting the OS of male patients the same as (a) and (b).

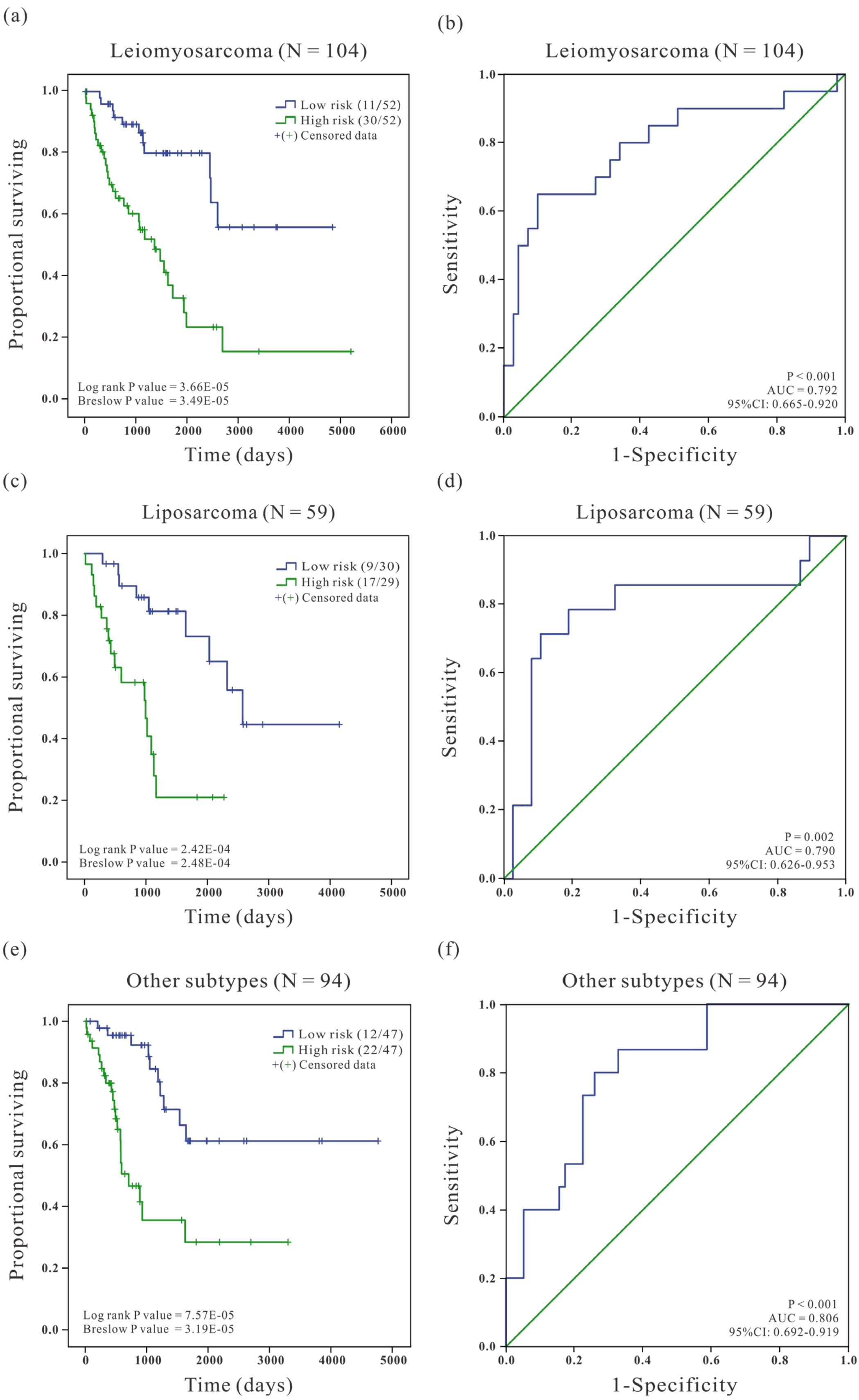

Supplementary Figure 5: Kaplan- Meier and ROC analysis for STS patients whose tumors belong to different histologic subtypes. (a) In the group of patients whose tumor subtype belong to leiomyosarcoma, Kaplan-Meier curve showed that the OS between the high- and low-risk patients is significantly different. (b) The ROC curve of the 3-DNA methylation biomarker is used to

demonstrate the sensitivity and specificity in predicting the OS of STS patients whose tumor subtype belong to leiomyosarcoma. (c) and (d) The Kaplan-Meier and ROC analysis of the biomarker in predicting the OS of patients whose tumors belong to liposarcoma. (e) and (f) The Kaplan-Meier and ROC analysis of the biomarker in predicting the OS of patients whose tumors belong to other subtypes, including fibromyxosarcoma, synovial sarcoma, giant cell sarcoma, etc.

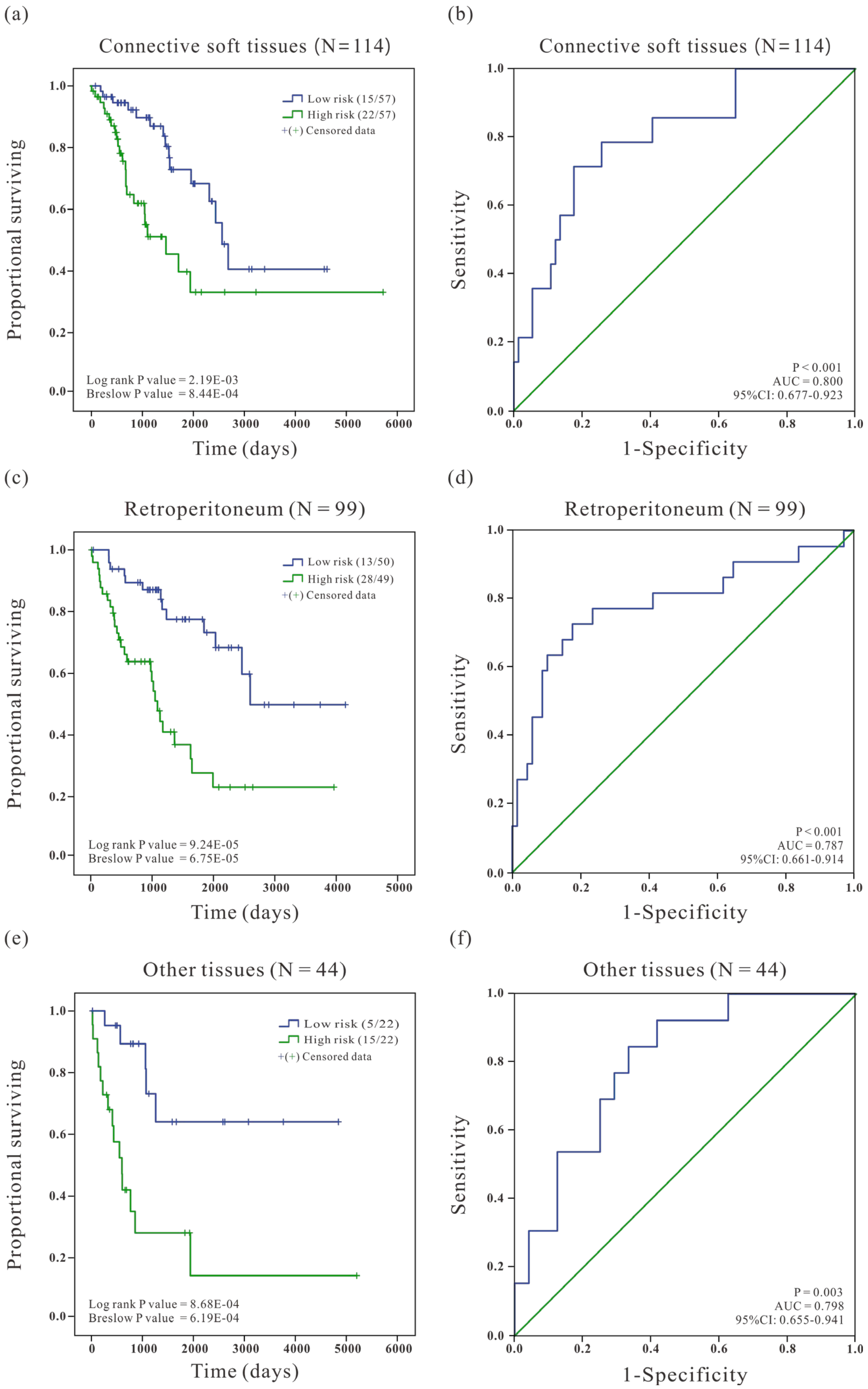

Supplementary Figure 6: Kaplan-Meier and ROC analysis for STS patients with tumor originate from different tissues. (a) The Kaplan-Meier curve of the biomarker in discriminating high- and low-risk patients with tumor originate from connective soft tissues. (b) The ROC curve showed the prognostic performance of this biomarker in STS patients with tumor originating from connective soft tissues. (c) and (d) The Kaplan-Meier and ROC analysis of the biomarker in patients with tumor originating from retroperitoneum. (e) and (f) The Kaplan-Meier and ROC analysis of the biomarker in patients with tumor originating from other tissues.

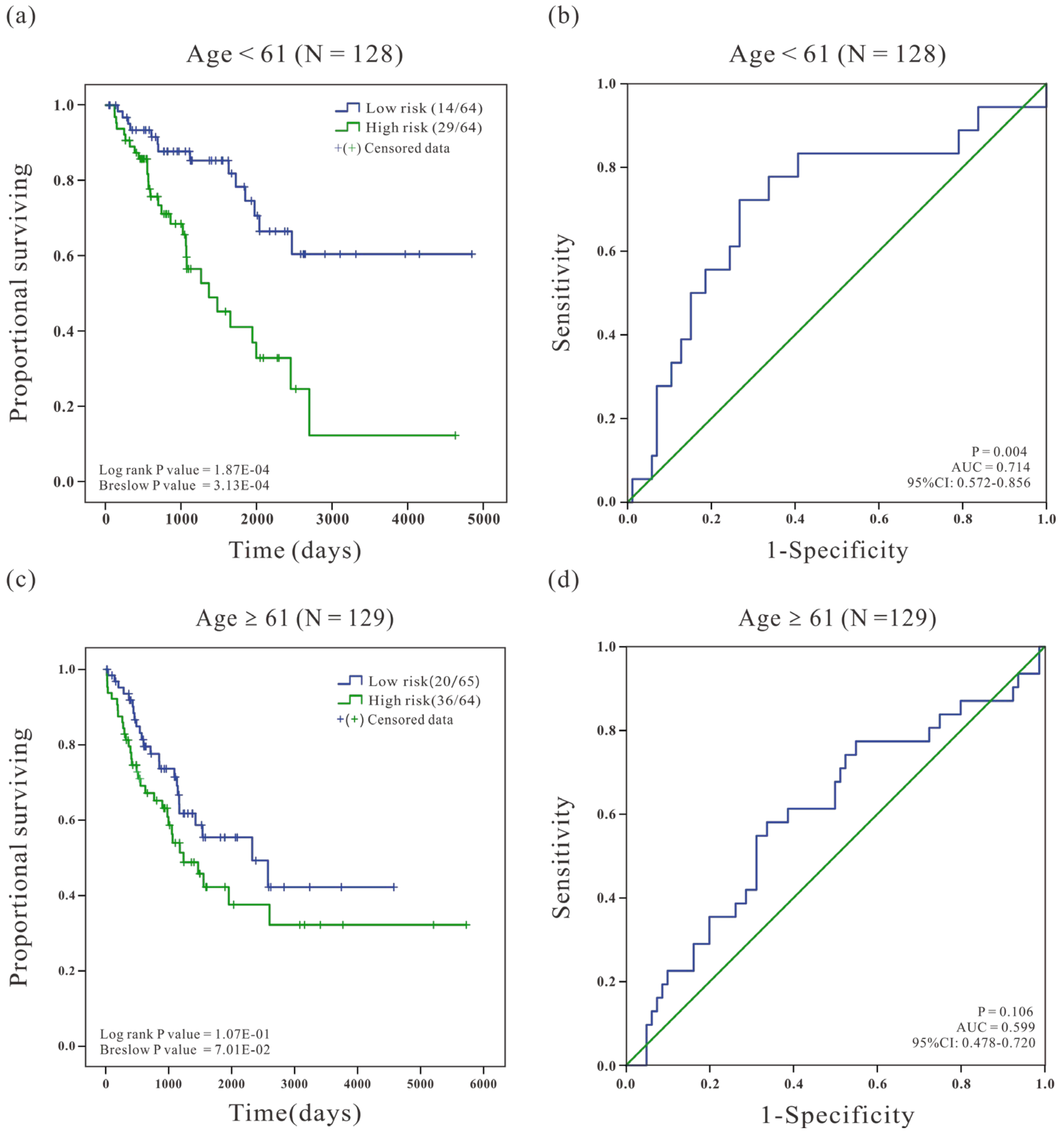

Supplementary Figure 7: Kaplan-Meier and ROC curves of the MST1 methylation biomarker in different age groups. (a) The Kaplan-Meier curve of the MST1 methylation biomarker in the group of patients aged less than 61 which estimated the survival difference between the low-risk group and the high-risk group. (b) The ROC curve of the MST1 methylation biomarker exhibits the prognostic performance in the group of patients aged less than 61. (c) and (d) are the Kaplan-Meier and ROC curves for patients group aged more than 61. It showed that in the group of patients aged more than 61, there was no survival difference between the low-risk group and the high-risk group.

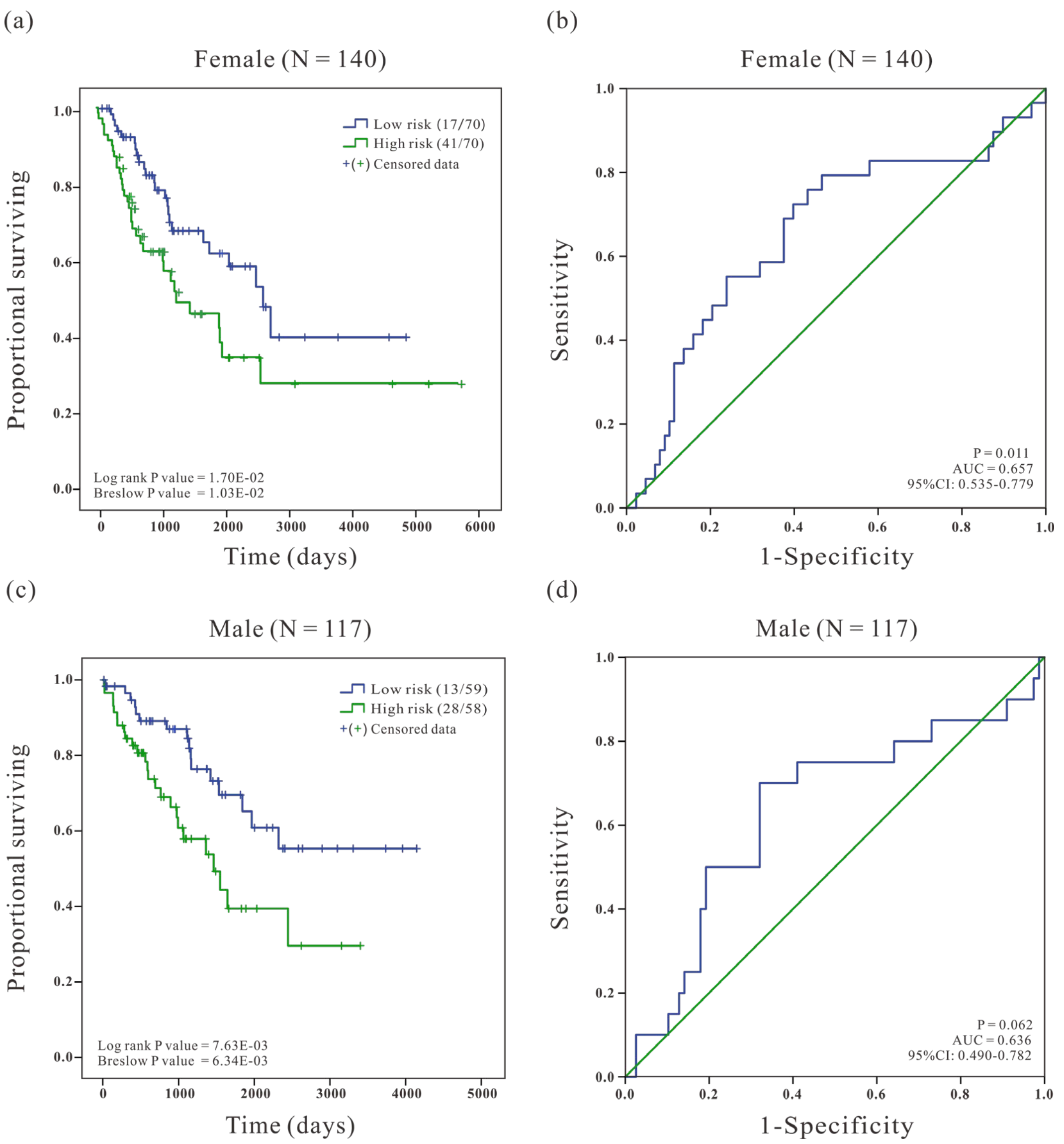

Supplementary Figure 8: Kaplan-Meier and ROC curves of the MST1 methylation biomarker for STS patients in patient groups with different gender. (a) In the female group, the Kaplan-Meier curve estimated the cumulative survival time of low- and high-risk STS patients. (b) In the female group, the ROC curve exhibited the prognostic performance of the MST1 methylation biomarker. (c) and (d) Kaplan-Meier and ROC analysis of the MST1 methylation biomarker in predicting the OS of male patients the same as (a) and (b).

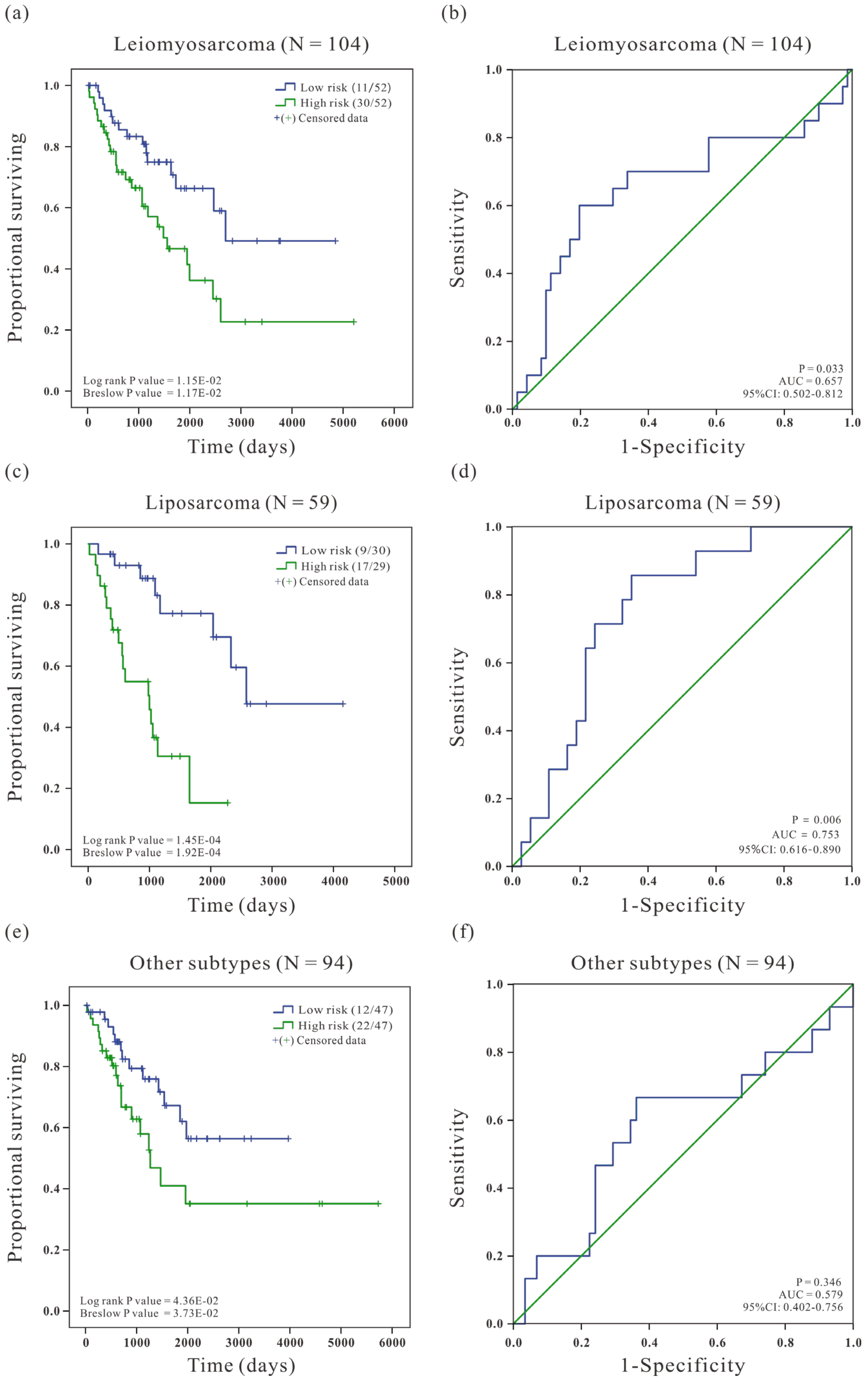

Supplementary Figure 9: Kaplan-Meier and ROC analysis of the MST1 methylation biomarker for STS patients whose tumors belong to different histologic subtypes. (a), (c) and (e) In the three groups, Kaplan-Meier curves showed that the OS between the high- and low-risk patients is different. (b), (d)

and (f) In the three groups, the ROC curves of the MST1 methylation biomarker used to demonstrate the sensitivity and specificity in predicting the OS of STS patients.

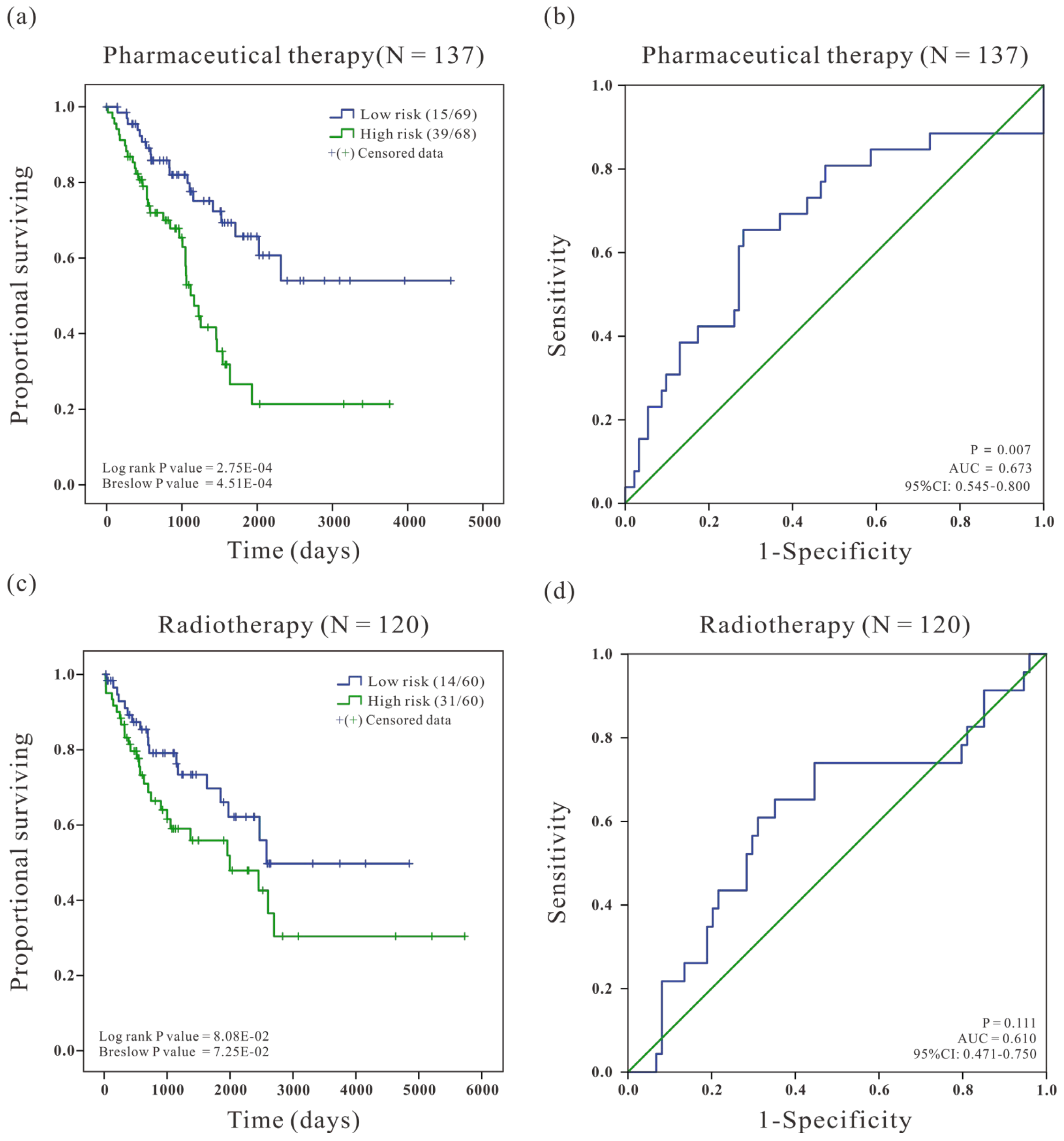

Supplementary Figure 10: ROC and Kaplan-Meier analysis of the MST1 methylation biomarker in groups of patients receiving different adjuvant therapies. (a) The Kaplan-Meier curve in the group of patients receiving adjuvant pharmaceutical therapy. (b) The ROC curve in the group of patients receiving pharmaceutical therapy. (c) The Kaplan-Meier curve in the group of patients receiving adjuvant radiotherapy. It showed that there was no survival difference between the low-risk group and the high-risk group. (d) The ROC curve in the group of patients receiving radiotherapy.

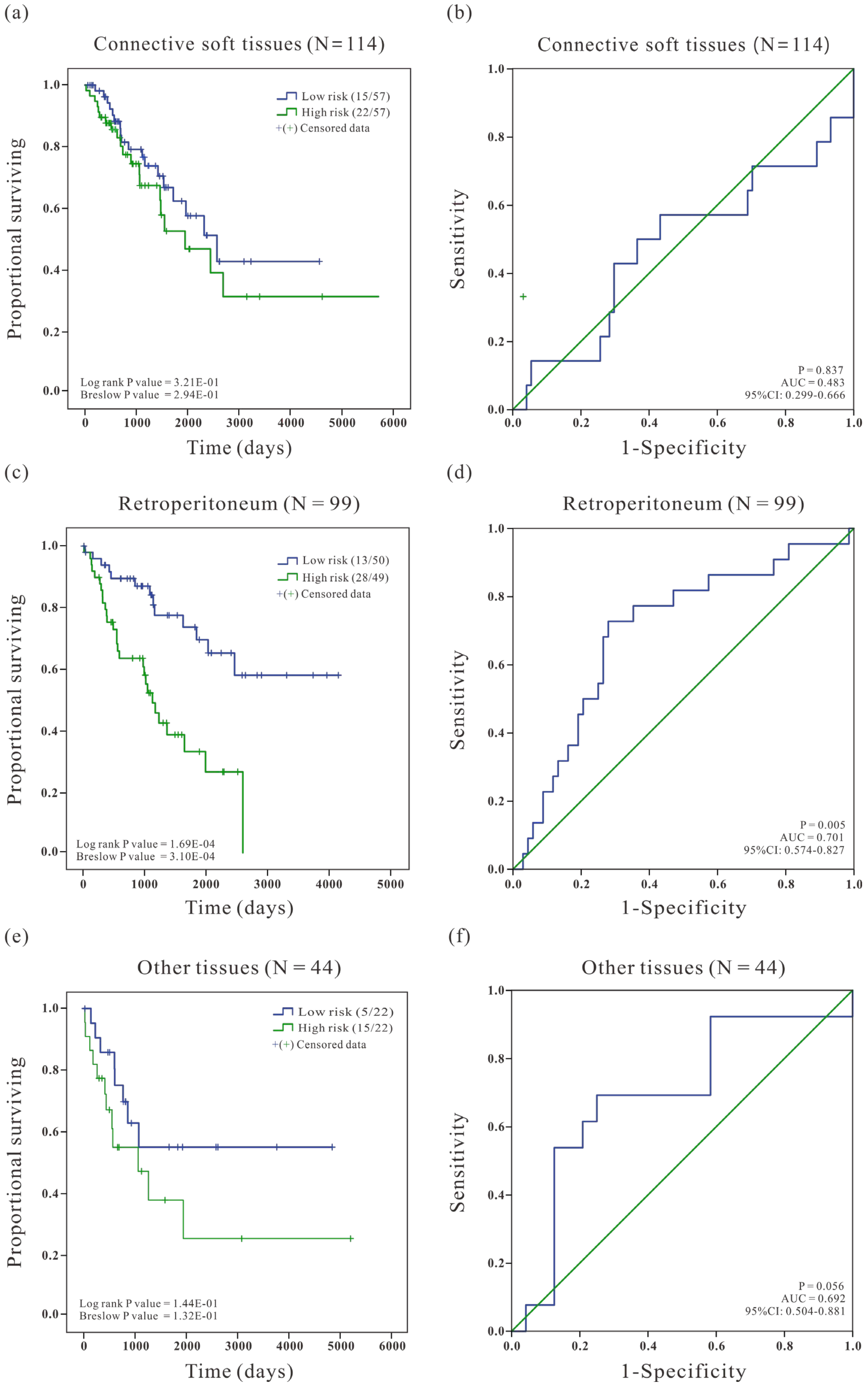

Supplementary Figure 11: Kaplan- Meier and ROC analysis for STS patients with tumor originate from different tissues. (a) The Kaplan-Meier curve of the MST1 methylation biomarker in discriminating high- and low-risk patients with tumor originate from connective soft tissue. It showed

that there was no survival difference between the low-risk group and the high-risk group. (b) The ROC curves of the MST1 methylation biomarker in patients with tumor originate from connective soft tissue. (c) and (d) are the Kaplan-Meier and ROC curves in patients with tumor originate from retroperitoneum. (e) and (f) are the Kaplan-Meier and ROC curves in patients with tumor originate from other tissues. In this group, the MST1 methylation biomarker cannot distinguish the high- and low-risk patients.
